# Effects of Tetrahydrocurcumin on Hypoxia-Inducible Factor-1*α* and Vascular Endothelial Growth Factor Expression in Cervical Cancer Cell-Induced Angiogenesis in Nude Mice

**DOI:** 10.1155/2015/391748

**Published:** 2015-02-18

**Authors:** Bhornprom Yoysungnoen, Parvapan Bhattarakosol, Suthiluk Patumraj, Chatchawan Changtam

**Affiliations:** ^1^Division of Physiology, Faculty of Medicine, Thammasat University, Rangsit Campus, Pathumthani 12120, Thailand; ^2^Department of Microbiology, Faculty of Medicine, Chulalongkorn University, Bangkok 10330, Thailand; ^3^Department of Physiology, Faculty of Medicine, Chulalongkorn University, Bangkok 10330, Thailand; ^4^Division of Physical Science, Faculty of Science and Technology, Huachiew Chalermprakiet University, Samut Prakan 10540, Thailand

## Abstract

Tetrahydrocurcumin (THC), one of the important *in vivo* metabolites of curcumin, inhibits tumor angiogenesis. Its effects on angiogenesis in cervical cancer- (CaSki-) implanted nude mice and its mechanisms on hypoxia-inducible factor-1*α* and vascular endothelial growth factor expression were investigated. Female BALB/c nude mice were divided into control (CON) and CaSki-implanted groups (CaSki group). One month after the injection with cervical cancer cells, mice were orally administered vehicle or 100, 300, and 500 mg/kg of THC daily for 30 consecutive days. The microvascular density (MVD) was evaluated using the CD31 expression. VEGF, VEGFR-2, and HIF-1*α* expression were also detected by immunohistochemistry. The MVD in CaSki + vehicle group was significantly increased compared to the CON + vehicle group. Interestingly, when treated with THC at all doses, the CaSki group showed a significant smaller number of the MVD. The CaSki + vehicle group also showed significantly increased VEGF, VEGFR-2, and HIF-1*α* expressions, but they were downregulated when mice were treated with THC at all doses. THC demonstrated an inhibitory effect against tumor angiogenesis in CaSki-implanted nude mice model. This effect is likely to be mediated by the downregulation of HIF-1-*α*, VEGF expression, and its receptor. THC could be developed into a promising agent for cancer therapy in the future.

## 1. Introduction

Cervical tumors frequently have a high vascular density which is responsible for spontaneous bleeding, a common symptom of cervical cancer. There is an urgent need for the development of novel agents to combat this type of cancer. Antiangiogenic agents have been identified as promising treatments that may enhance therapeutic outcomes when used in combination with chemotherapy. Currently, there are a number of biological agents in clinical development that inhibit tumor angiogenesis by targeting vascular endothelial growth factor (VEGF) and its signaling.

VEGF, also known as vascular permeability factor (VPF), is a major mediator of tumor angiogenesis and is tightly controlled by oxygen tension [1]. Hypoxia induces the expression of VEGF and its receptor via hypoxia inducible factor-1*α* (HIF-1*α*) [2]. VEGF promotes mobilization of endothelial progenitor cells, cell proliferation, migration, and survival [3]. VEGF can also induce the leakage of blood vessels which contributes to an increasing edema around the tumor tissue [4]. With the effect of VEGF, blood vessels become incapable of providaing efficient blood flow, thus creating the hypoxic condition to the tumor [5] which in turn stimulates the continuous production of VEGF. This continuous cycle of events inevitably leads to tumor angiogenesis and tumor progression under hypoxic conditions [6]. VEGF was thus found to be associated with a poor prognosis of cancer [7, 8]. Our recent study also showed that VEGF is overexpressed in cervical cancer- (CaSki-) xenografts in nude mice [9].

VEGF binding sites were identified on vascular endothelial cells and called VEGFR-1 (Flt-1) [10] and VEGFR-2 (Flk-1/KDR) [11]. VEGFR-2 has been found to play a pivotal role in the activation of downstream components that are responsible for proliferation, including endothelial cell invasion, migration, differentiation, and embryonic angiogenesis [12, 13]. Recently, Kim et al. indicated that VEGF and VEGFR-2 contributed to angiogenesis through the migration of stem cells [14]. Thereby, targeting the major proangiogenic stimulus VEGF and its receptor became the focus for therapeutic intervention.

Curcumin (diferuloylmethane) (CUR) is a phenolic compound from the plant* Curcuma longa*. Pharmacological outcomes of CUR have been widely reported. Our earlier study showed that CUR had anticancer and antiangiogenic effects in cervical cancer xenografts in nude mice [9]. However, the significant reduction in tumor progression and tumor angiogenesis was observed only within mice treated with high doses of CUR. This finding might be due to the pharmacokinetic features of CUR which has poor oral bioavailability. Tetrahydrocurcumin (THC) is one of the major metabolites of CUR with phenolic and *β*-diketo moieties similar to CUR [15]. We also demonstrated that THC showed more potent tumor antiangiogenesis activity than CUR [16], and this might be due to its possessing higher antioxidant activity. However, the effect of THC on tumor angiogenesis especially in a cervical cancer- (CaSki-) implanted nude mice model has not yet been reported. Therefore, the present study was designed to determine the effects of THC on angiogenesis in cervical cancer- (CaSki-) implanted nude mice and to study the possible mechanisms of THC on HIF-1*α* and VEGF/VEGFR-2 pathway.

## 2. Methods

### 2.1. Cell Line and Cell Culture

Cervical cancer cells (CaSki) were purchased from the American Type Culture Collection. The cell lines were cultured in an MEM medium supplemented with 10% fetal bovine serum. All cultures were maintained in an incubator at 37°C with 5% CO_2_ in a humidified atmosphere.

### 2.2. CaSki-Induced Tumor Mice

BALB/c-nude female mice weighing about 20–25 g were used. The animal experiments were conducted according to the guidelines on experimental animals of The National Research Council of Thailand (1999). The mice were divided into 6 groups: (1) controls supplemented with corn oil (Control + vehicle; *n* = 6), (2) controls supplemented with THC (500 mg/kg) (Control + THC; *n* = 6), (3) CaSki-implanted mice supplemented with corn oil (CaSki + vehicle; *n* = 6), (4) CaSki-implanted mice supplemented with THC (100 mg/kg) (CaSki + THC100, *n* = 6), (5) CaSki-implanted mice supplemented with THC (300 mg/kg) (CaSki + THC300, *n* = 6), and (6) CaSki-implanted mice supplemented with THC (500 mg/kg) (CaSki + THC500, *n* = 6).

For the CaSki groups, a suspension of 10 × 10^6^ CaSki cells in 0.2 mL MEM [17] was subcutaneously injected into the dorsa of mice at the proximal midline while the control group was injected with MEM. The tumors were measured with Vernier calipers every 3-4 days by using the formula *a* ^2^ × *b* × 0.52 (where *a* is the shortest and *b* is the longest diameter). When the tumor volume was 100–120 mm^3^, the mice were randomized. Following this, the mice were supplemented daily with vehicle or THC at the doses of 100, 300, or 500 mg/kg body weight for one month.

### 2.3. Study of Tumor Microvasculature

On study day 30, the mice were anaesthetized with sodium pentobarbital (50 mg/kg bw,* i.p*). Fluorescence tracers 0.1 mL of 0.5% fluorescein isothiocyanate- (FITC-) labeled dextran (MW = 200,000, Sigma Chemical, USA) were injected into the jugular vein. The tumor microvasculature was visualized under a confocal microscope.

### 2.4. Immunohistochemistry for CD31 Expression and Microvessels Density (MVD) Determination

After the microvascular study, the mice were sacrificed and the tumors were fixed in 10% formalin. Immunohistochemistry was performed using 5 *μ*m thick paraffin sections. Paraffin sections were dewaxed and rehydrated through xylene and a graded alcohol series. Endogenous peroxidase activity was blocked with 3% hydrogen peroxide for 15 min at room temperature. After washing in water, nonspecific binding sites were blocked with 5% bovine serum in phosphate-buffered saline (PBS) for 30 min at room temperature. The tissue slide samples were incubated with primary monoclonal antibody CD31 (Thermo Fischer Scientific, UK) (1 : 500) at 4°C overnight. The slide was then gently rinsed with PBS and developed by the Envision system/HRP (DAKO cytomation, USA) for 30 min and substrate-chromogen for 10 min at room temperature. The nuclei were counterstained with Mayer's hematoxylin.

To quantify angiogenesis, microvessel density (MVD) was assessed by immunostaining with the anti-CD31 antibody as previously described [18]. The sections were observed first under low power (×40), then the densest area of microvessel sections was selected and counted under high power (×200, the surface area of every vision field being 0.4 mm^2^).

### 2.5. Immunohistochemistry for VEGF, VEGFR-2, and HIF-1*∞* Expression

The tissue slide samples were incubated with primary monoclonal antibody VEGF (Thermo Fischer Scientific, UK) (1 : 75), antibody VEGFR-2 (1 : 250) (GeneTex Inc., USA), and antibody HIF-1*∞* (1 : 1,000) (GeneTex Inc., USA) at 4°C overnight. The slide was then gently rinsed with PBS and developed by the Envision system/HRP (DAKO cytomation, USA) for 30 min and substrate-chromogen for 10 min at room temperature. The nuclei were counterstained with Mayer's hematoxylin.

### 2.6. Staining Analysis

The sample image from each slide was subtracted from the corresponding background image. The image threshold was standardized to delineate the labeled structures and then applied to all images of an individual experiment. The staining intensity and the percentage of positive stained VEGF, VEGFR-2, and HIF-1*∞* were analyzed. Staining intensity (*I*) was scored as 0 (none), 1+ (weak), 2+ (moderate), and 3+ (strong). The percentage of positive stains (*P*) was scored as 0, 0% immunopositive cells; 1, ≤25% positive cells; 2, 26–50% positive cells; 3, ≥51–75% positive cells; 4, ≥76%. The sum of both (*I*) and (*P*) scores was evaluated for each case and a final score was assigned 0 (negative), 1–3 (weak expression), 4-5 (moderate expression), and 6-7 (strong expression) [19].

### 2.7. Statistical Analysis

Data were expressed as means with standard error. SPSS.13 software was used for statistical analysis. Student's unpaired *t*-test was applied for comparison of the means of two groups (Control and CaSki + vehicle groups), and analysis of variance was used for the means of multiple groups. The correlation between CD31 expressions and VEGF, VEGFR-2, and HIF-1*∞* was assessed with Pearson correlation test. For all of the value differences, *P* value less than 0.05 was considered significant.

## 3. Results

### 3.1. Antiangiogenic Effect of THC in CaSki-Implanted Mice

**Figure 1:**
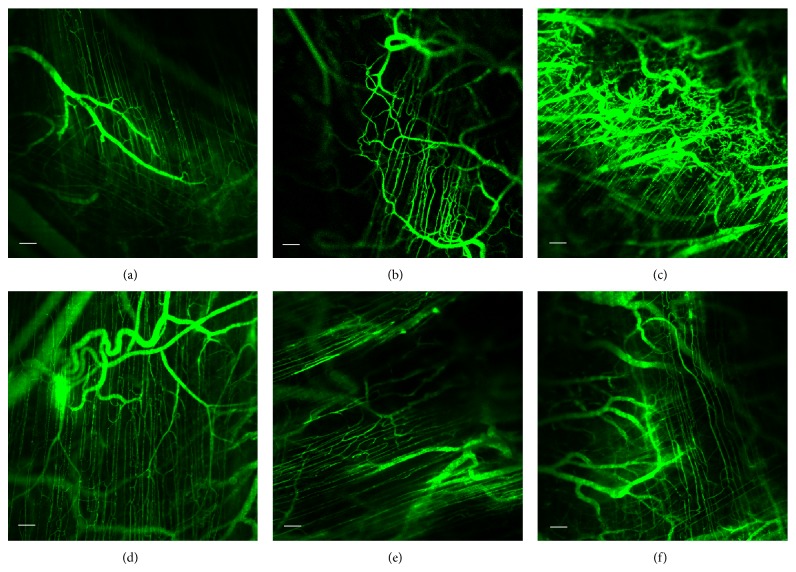
Confocal images of tumor microvasculature in Control + vehicle group (a), Control + THC500 group (b), CaSki + vehicle group (c), CaSki + THC100 group (d), CaSki + THC300 group (e), and CaSki + THC500 (f), Bar = 100 *µ*m, 10x.

The confocal fluorescent images of the microvasculature for controls (a and b), CaSki + vehicle group (c), and CaSki + THC groups (d–f) are demonstrated in Figure 1. There was a marked increase in capillary networks in the CaSki-groups. These networks were heterogeneous, tortuous, dilated, and displayed hyperpermeability with extravasations of fluorescence tracer. However, the appearance of neocapillaries induced by CaSki was markedly reduced after receiving all doses of THC (100, 300, and 500 mg/kg). In addition, the abnormalities of the neocapillary network pattern were attenuated by THC.

**Figure 2:**
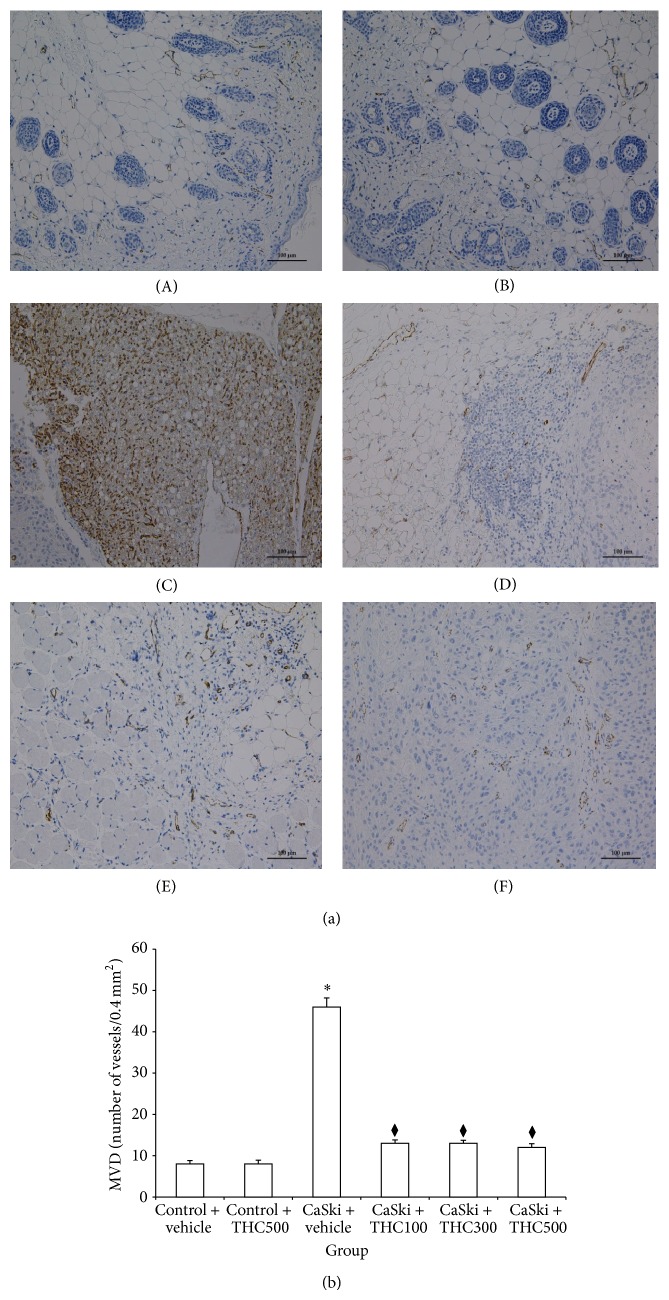
(a) CD-31 expression in Control + vehicle group (A), Control + THC group (B), CaSki + vehicle group (C), CaSki + THC100 group (D), CaSki + THC300 group (E), and CaSki + THC500 group (F), Bar = 100 *µ*m, 200x. (b) Microvascular density (numbers/0.4 mm^2^) (mean ± SEM). ^*^ *P* < 0.001 versus Control + vehicle group, ^*♦*^ *P* < 0.001 versus CaSki + vehicle group.

A detection of the CD31 antigen expression was used to determine the quantity of the angiogenesis process. CD31 primarily indicated the presence of endothelial cells in histological tissue sections.  Figure 2(a) shows representative immunostaining for CD31 in control ((A) and (B)), CaSki + vehicle (C), and CaSki + THC ((D)–(F)) groups. In normal skin tissue, few CD31 expressions were detected adjacent to sweat glands, whereas they were highly expressed in CaSki-implanted tissues. However, all doses treated with THC attenuated the CD31 expression.

We measured the microvascular density (MVD), as determined by CD31 staining, to assess the quantity of the angiogenesis process. In Figure 2(b), the MVD of both CON + vehicle and CON + THC500 were similar (8 ± 0.84 and 8 ± 0.95, resp.). The MVD was significantly higher in the CaSki + vehicle group than in the control group (46 ± 2.23 versus 8 ± 0.84; *P* < 0.001). The MVD was significantly decreased by all doses of treatments with THC (100, 300, and 500 mg/kg) (13 ± 0.85, 13 ± 0.74, and 12 ± 0.91, resp.).

### 3.2. Effects of THC on VEGF and Its Receptor

**Figure 3:**
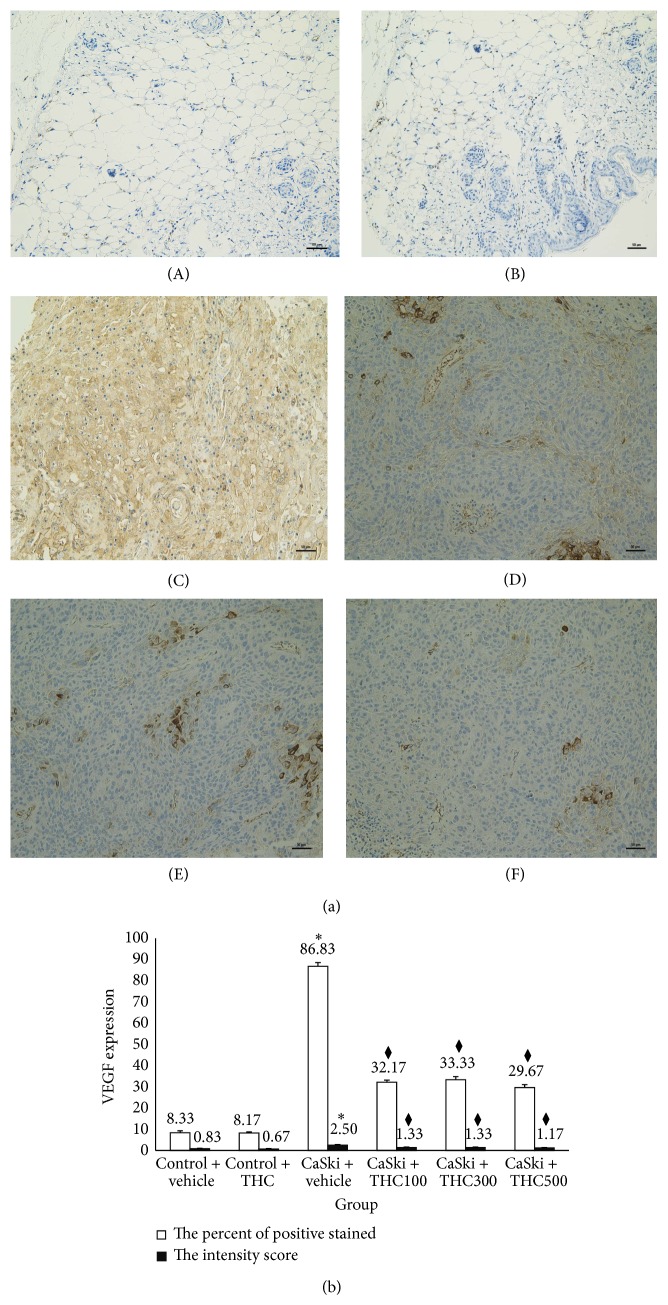
(a) VEGF expression in Control + vehicle group (A), Control + THC group (B), CaSki + vehicle group (C), CaSki + THC100 group (D), CaSki + THC300 group (E), and CaSki + THC500 group (F), Bar = 50 *µ*m, 200x. (b) VEGF expression (mean ± SEM). ^*^ *P* < 0.001 versus Control + vehicle group, ^*♦*^ *P* < 0.001 versus CaSki + vehicle group.

**Figure 4:**
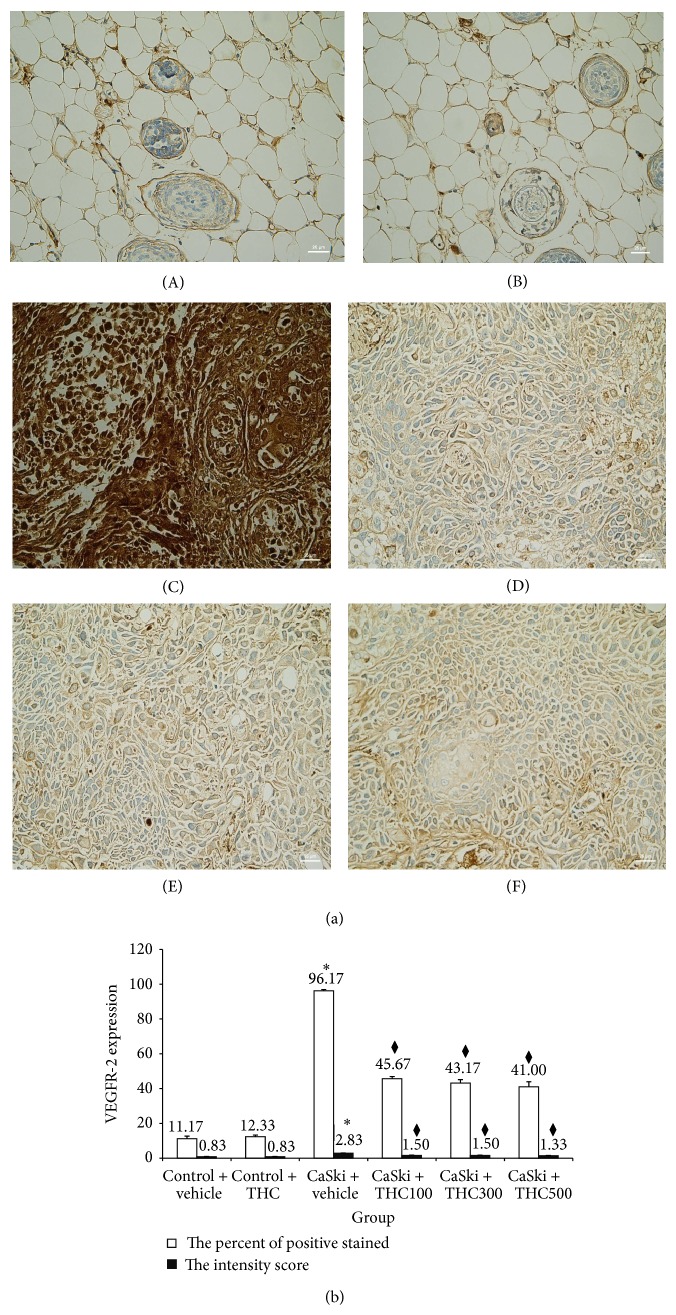
(a) VEGFR-2 expression in Control + vehicle group (A), Control + THC group (B), CaSki + vehicle group (C), CaSki + THC100 group (D), CaSki + THC300 group (E), and CaSki + THC500 group (F), Bar = 25 *µ*m, 400x. (b) VEGFR-2 expression (mean ± SEM). ^*^ *P* < 0.001 versus Control + vehicle group, ^*♦*^ *P* < 0.001 versus CaSki + vehicle group.

Figures 3(a) and 4(a) show microscopic images of immunohistochemical stained sections for VEGF and VEGFR-2 expression, respectively. Immunoreactivity for VEGF protein (shown in brown) was presented diffusely in the cytosolic of cancer cells (Figure 3(a)). VEGFR-2 expression was mainly localized adjacent to the plasma membrane (Figure 4(a)). Some VEGFR-2 was also distributed intracellularly. Stronger VEGF and VEGFR-2 expression was found in the CaSki + vehicle group than in the control group. Interestingly, our study demonstrated that THC attenuated VEGF and VEGFR-2 expression.

Figures 3(b) and 4(b) show the percentage of positive staining of VEGF and VEGFR-2, respectively. In the CaSki + vehicle group, VEGF (86.83 ± 1.74%) and VEGFR-2 (96.17 ± 0.70%) expression were significantly higher than in the control group (VEGF: 8.33 ± 0.88%; VEGFR-2: 11.17 ± 1.45%) (*P* < 0.001).

In all CaSki + THC groups, the percentage of positive staining of VEGF was significantly lower than in the CaSki + vehicle group (*P* < 0.001). VEGF in CaSki + THC100 was 31.17 ± 0.95%, in CaSki + THC300 was 33.33 ± 1.45%, and in CaSki + THC500 was 29.67 ± 1.33%. In the same fashion, the percentage of positive staining of VEGFR-2 was also attenuated by the treatments with THC (45.67 ± 1.17%, 43.17 ± 1.92%, and 41.00 ± 2.86% at the doses of 100, 300, and 500 mg/kg, resp. (*P* < 0.001)).

The intensity scores of VEGF and VEGFR-2 expression are shown in Figures 3(b) and 4(b). The CaSki + vehicle group showed strong intensity scores for VEGF (mean score = 2.50 ± 0.22) and VEGFR-2 (mean score  = 2.83 ± 0.17) whereas the control group showed weak intensity scores (mean score = 0.83 ± 0.17 and 0.83 ± 0.17 for VEGF and VEGFR-2, resp.).

Staining intensity scores for VEGF expression were significantly reduced in CaSki + THC100 (mean score = 1.33 ± 0.21), CaSki + THC300 group (mean score = 1.33 ± 0.21), and CaSki + THC500 group (mean score  = 1.17 ± 0.17) (*P* < 0.001). Similarly, staining intensity scores for VEGFR-2 expression were significantly reduced in all treated groups which were 1.50 ± 0.22, 1.50 ± 0.22, and 1.33 ± 0.21 in CaSki + THC300, CaSki + THC500, and CaSki + THC300 groups, respectively (*P* < 0.001).

The total scores for VEGF positive staining and intensity revealed that the CaSki + vehicle group had strong VEGF expression (mean total score = 6.50 ± 0.21) whereas the control group had weak VEGF expression (mean total score = 1.83 ± 0.17). The total scores for VEGFR-2 in the CaSki + vehicle group also had strong VEGFR-2 expression (mean total score  = 6.80 ± 0.17) whereas the control group had weak VEGFR-2 expression (mean total score = 1.83 ± 0.17).

In the treated groups, the total scores for VEGF expression revealed weak expressions in CaSki + THC100 (mean total score = 3.33 ± 0.21), CaSki + THC300 (mean total score = 3.33 ± 0.21), and CaSki + THC500 groups (mean total score = 3.17 ± 0.17). The total scores for VEGFR-2 expression also showed weak expression in all treated groups with the mean total scores of CaSki + THC100, CaSki + THC300, and CaSki + THC500 at 3.50 ± 0.22, 3.50 ± 0.22, and 3.00 ± 0.00, respectively.

### 3.3. Effects of THC on HIF-1*α*

**Figure 5:**
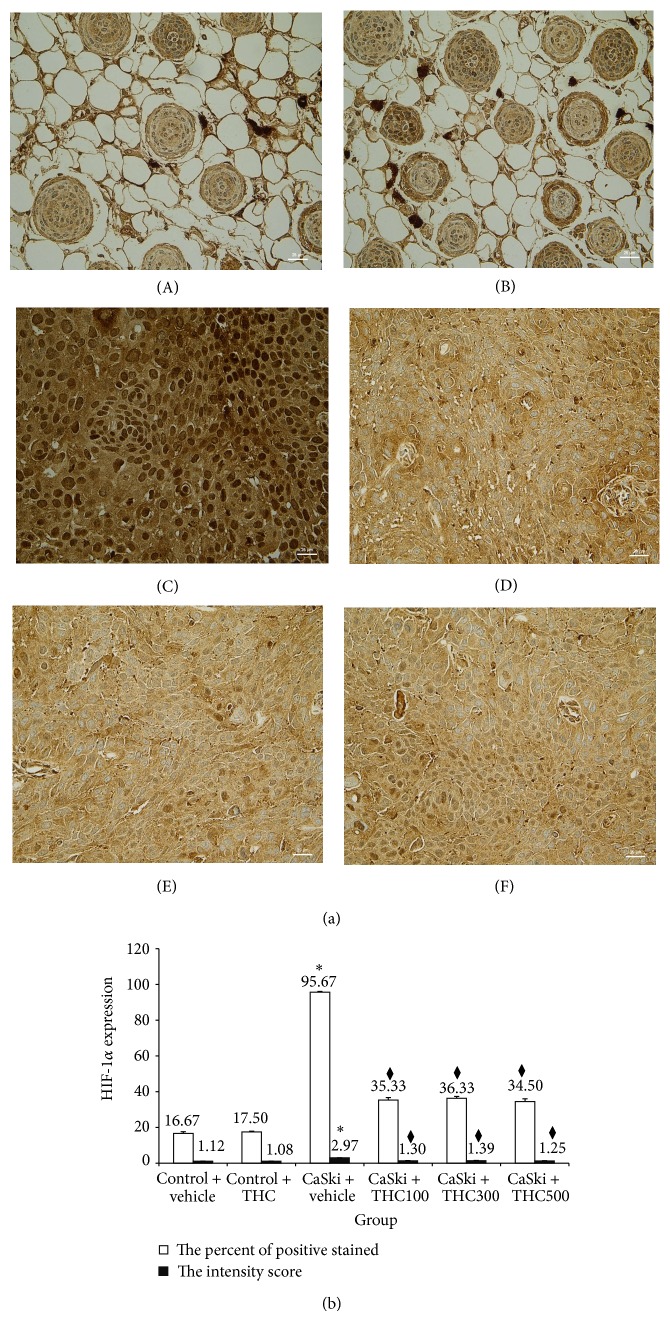
(a) HIF-1*α* expression in in Control + vehicle group (A), Control + THC group (B), CaSki + vehicle group (C), CaSki + THC100 group (D), CaSki + THC300 group (E), and CaSki + THC500 group (F), Bar = 25 *µ*m, 400x. (b) HIF-1*α* expression in Control + vehicle group (A), Control + THC group (B), CaSki + vehicle group (C), CaSki + THC100 group (D), CaSki + THC300 group (E), and CaSki + THC500 group (F), Bar = 25 *µ*m, 400x.

Overall, an increase in both nuclear and cytoplasmic HIF-1*α* expression was found in the CaSki + vehicle group (Figure 5(a)). Again, our study demonstrated that THC attenuated HIF-1*α* expression. The quantitative data showed the percentage of positive staining of HIF-1*α* expression significantly increased in the CaSki + vehicle group (95.67 ± 0.42%) compared to the control group (16.67 ± 0.95%) (*P* < 0.001) (Figure 5(b)). The percentage of positive staining of HIF-1*α* was attenuated by the treatments with THC at the doses of 100, 300, and 500 mg/kg which were 35.33 ± 1.38%, 36.33 ± 1.05%, and 34.50 ± 1.48%, respectively. Significant reductions in HIF-1*α* positive staining were found in all treated groups (*P* < 0.001).

The intensity score of HIF-1*α* expression in CaSki + vehicle group was high (mean score = 2.97 ± 0.03) but was low (mean score = 1.12 ± 0.05) in the control group (Figure 5(b)). However, staining intensities for HIF-1*α* expression were significantly reduced in CaSki + THC100 (mean score = 1.30 ± 0.19), CaSki + THC300 (mean score = 1.39 ± 0.19), and CaSki + THC500 (mean score = 1.25 ± 0.17) groups (*P* < 0.001).

The total score for HIF-1*α* expression showed that the CaSki + vehicle group had strong HIF-1*α* expression (mean total score = 6.63 ± 0.33) whereas the control group had weak HIF-1*α* expression (mean total score = 2.12 ± 0.05). The HIF-1*α* expression extent and intensity scores in the treated group revealed that weak expressions were found in CaSki + THC100 (mean total score = 3.30 ± 0.19), CaSki + THC300 (mean total score = 3.39 ± 0.19), and CaSki + THC500 groups (mean total score = 3.25 ± 0.17), respectively.

### 3.4. The Relationship between the Expression of CD31, VEGF, VEGFR-2, and HIF-1*α* Expression

The percentage of stained positive cells for both VEGF and VEGFR-2 expression was positively correlated with MVD in CaSki-implanted mice (*r* = 0.96 and *r* = 0.92, resp., *P* < 0.001) as well as the respective intensity scores (*r* = 0.78 and *r* = 0.82, resp., *P* < 0.001). Furthermore, the percentage of positive cells and intensity scores for VEGF expression were positively correlated with both VEGFR-2 (*r* = 0.98 and *r* = 0.65, resp. (*P* < 0.001)) and HIF-1*α* expression (*r* = 0.99 and *r* = 0.81, resp. (*P* < 0.001)).

## 4. Discussion

In the present study, we determine the effects of THC on tumor angiogenesis in cervical cancer- (CaSki-) implanted nude mice and study the mechanisms of THC on hypoxia-inducible factor-1*α* and VEGF/VEGFR-2 pathway. By using confocal fluorescent microscopy, implanted CaSki cells caused changes in the microvascular network, including the appearance of dilatation, tortuosity, and hyperpermeability with extravasations of fluorescence tracer from the microvessels. These changes could be a result of angiogenic growth factors, in particular, VEGF.

VEGF plays a pivotal role in the control of angiogenesis as well as tumor growth and metastasis [12, 20]. Overexpression of VEGF has been associated with tumor progression and poor prognosis in several tumors, including cervical cancer [7]. It is possible that VEGF facilitates tumor progression by inducing the disorganization, hyperpermeability, and tortuosity of vasculatures, which are all conditions favoring malignant cell invasion. In support of this hypothesis, increases of MVD and tortuosity have been observed in our CaSki-implanted model. Moreover, perfusion of VEGF-induced vessels could act as a chemoattractant for tumor cell migration and eventually lead to tumor cell invasion along the vascular system [21]. In the present study we found VEGF overexpression in CaSki-implanted mice, and we demonstrated that VEGF expression and MVD were strongly correlated in the cancer tissues (*r* = 0.96). These results were similar to our previous finding that VEGF played an important role in tumor biological behavior and neovascularization in cervical cancer-implanted mice models [9].

The expression of VEGF is controlled by HIF-1*α* under hypoxia conditions [22]. HIF-1 is a transcription factor that mediates cellular and systemic homeostatic responses to reduced O_2_ availability in mammals, including angiogenesis, erythropoiesis, and glycolysis. During tumor progression, the tumor mass develops hypoxic zones where HIF-1*α* can induce the accumulation of VEGF proteins [23]. In the present study, we demonstrate that HIF-1*α* significantly increases in CaSki-implanted mice. Moreover, we also found that HIF-1*α* expression strongly correlated with VEGF expression (*r* = 0.99). Lee and his coworkers demonstrated that a hypoxic zebrafish tumor model offers hypoxia-induced angiogenesis in promoting tumor cell dissemination and metastasis [21]. However, the mechanisms underlying the hypoxia-induced angiogenesis in mediation of tumor cell dissemination and metastasis remained elusive. Our results provide compelling evidence that hypoxia-induced angiogenesis are mediated by VEGF-induced pathological blood vessels.

Once VEGF binds to its high affinity receptors (Flt-1/VEGFR-1, Flk-1/KDR/VEGFR-2), it promotes a signaling cascade that ultimately produces increased endothelial cell survival, proliferation, vascular permeability, migration, and invasion [12, 20]. Although VEGFR-1 and VEGFR-2 have a similar structure, they have different biological roles. For instance, while VEGFR-1 has no role in endothelial cell proliferation [24], VEGFR-2 plays a pivotal role in the activation of downstream components that are responsible for proliferation, including endothelial cell invasion, migration, differentiation, and embryonic angiogenesis as well as tumor angiogenesis [25, 26]. VEGFR-2 preferentially utilizes the PLC*γ*-PKC-MAPK pathway for signaling [27]. The present study shows that strong expression of VEGFR-2 was found in the CaSki + vehicle group. Moreover, a high correlation between VEGFR-2 and VEGF was found in CaSki-induced tumor angiogenesis in nude mice (*r* = 0.98). Therefore, the VEGF-VEGFR-2 system is an important target for antiangiogenic therapy in cancer.

Antiangiogenic therapy of cancers offers the possibility of a low toxicity treatment without acquisition of drug resistance due to the genetic stability, homogeneity, and low mutational rate of endothelial cells [28]. The current study revealed that all doses (100, 300, and 500 mg/kg) of THC decreased microvascular density. Furthermore, the pathological features including host-microvascular dilatation, tortuosity, and hyperpermeability, induced by tumor were attenuated by THC treatments (Figure 1). This indicates that THC treatment is a means of controlling rather than destroying the angiogenic process which leads to a process of vascular normalization.

Our previous study found that CUR was effective at doses of 1,000 mg/kg to inhibit tumor growth and angiogenesis in CaSki-implanted mice models [9]. Interestingly, the present study showed that THC was effective at doses of 100 mg/kg, ten times less than CUR. THC could decrease both VEGF and VEGFR-2 expression during angiogenesis (Figures 3(a) and 4(a)) and suppress HIF-1*α* expression (Figure 5(a)). This suggests that THC is more effective to inhibit angiogenesis than its parent compound, CUR.

THC is one of the major metabolites of CUR* in vivo*, and it may play a crucial role in CUR-induced biological effects. In contrast to CUR, THC is stable in phosphate buffer and in saline at various pH values [29]. Interestingly, THC is easily absorbed through the gastrointestinal tract, suggesting that THC is a preferred potential candidate for the development of an anticancer agent. In this study, we confirmed that THC dramatically reduced microvascular density, HIF-1*α*, and VEGF as well as VEGFR-2 protein expression. These results clearly suggest that THC inhibits tumor angiogenesis by downregulation of HIF-1-*α*, VEGF/VEGFR-2 pathway.

## 5. Conclusion

Our study shed new light on our understanding of the mechanisms of THC in cervical cancer intervention. We demonstrated that THC, one of the active anticancer forms of CUR* in vivo*, markedly inhibited tumor angiogenesis in CaSki-implanted female nude mice models by downregulation of HIF-1-*α*, VEGF/VEGFR-2 pathway. THC should be further investigated for its potential as a candidate for the development of an additional and/or alternative agent for the treatment of human cervical cancer.
